# Physical Exercise: An Overview of Benefits From Psychological Level to Genetics and Beyond

**DOI:** 10.3389/fphys.2021.731858

**Published:** 2021-08-12

**Authors:** Yucong Wang, Kalaiselvan Ashokan

**Affiliations:** ^1^Department of Joint Surgery, Ningbo No. 9 Hospital, Ningbo, China; ^2^Department of Biochemistry and Hematology, MV Hospital for Diabetes and Prof. M. Viswanathan Diabetes Research Centre, Chennai, India

**Keywords:** exercise, genetics, physical activity, benefits, gene

## Abstract

Any form of physical activity, including exercise, is linked with preventing several diseases including metabolic disorders, cancer, and mood disorders. Beyond benefits, its therapeutic activity is inconclusive in terms of types, intensity, and individual training status, and this could be a major research for prescribing exercise as a therapeutic strategy. Exercise and its myriad forms occupy the space on clinical recommendation, which implies that quantifiable benefits of the same have been proven. Further, the benefits of exercise and its impact have also been found to have a genetic underlying interaction, which has created a niche of personal genomics, wherein apart from diet, an exercise regimen also becomes tailorable for every individual. Many subjective well-being reports highlighted daily exercise to keep mental and general health in excellent conditions, and the uncertainties around it. Thus, adopting an exercise behavior and inculcating it as a routine has been recommended. Further, the kind of benefit that can be extracted out of exercise and training is to a great extent influenced by genetic markers around fat, obesity, hunger, as well as satiety. Genetic markers can also impact the body temperature during exercise, making the entire experience of training either comfortable or unpleasant. Thus, there is an overwhelming amount of scientific evidence that has gathered around the benefits of exercise, which has become a pressing need from the 21st century when the belief in the value of exercise started waning, and that spiked up the era of lifestyle and noncommunicable ailments.

## Introduction

Physical exercise has a proven and documented effect on mortality, wherein its preventive impact on diseases like cancer has entitled it to be therapeutic, beyond a healthy habit. For example, exercise has been demonstrated to cause 60% reduction in tumor incidence and growth in several mouse models, and this may be due to exercise-induced influx of immune cells in tumors (Kujala et al., 1998; Idorn and Straten, 2017). The longevity benefit of exercise has also been proven in large cohort studies that found adherence to physical activity (PA) recommendations to be beneficial (McGLory et al., 2018). Further, the exercise-induced longevity benefits threshold is obtained at a level three to five times higher (450–750 min/week) over the minimum recommendations among adults (Arem et al., 2015). The first PA recommendation guidelines were released in the year 2008 by the federal government, which recommends that a 150–300 min of moderate-intensity or 75–150 min of vigorous-intensity of aerobic exercise can provide substantial health benefits (Physical Activity Guidelines Advisory Committee). Studies have also assessed the impact of environment toward the development of exercise tendency in childhood, wherein influence of parents, peers, and coaches have been documented toward contribution for a motivational climate (Allen and Hodge, 2006; Keegan et al., 2009). Studies have also discussed intrinsic and extrinsic motivation models, wherein parents are the global influence, whereas peers and coaches exert motivation in both contextual and situational levels (Vallerand, 1997). Exercise has also been reviewed and proven as a promising adjunct intervention for mood disorders, including bipolar and major depressive disorder (MDD) (Hearing et al., 2016). However, several factors of exercise are inconclusive such as type, intensity, duration, and training status of the individual, thus recommending exercise as major therapeutics to prevent or control diseases has bigger challenges. Therefore, this review focuses on the benefits of exercise from psychological response to genetics.

## Psychological Impact of Exercise

Exercise has been evaluated as an adjunct intervention for mood disorders including MDD and bipolar disorder. The Healthy Body Healthy Mind Feasibility Study involved engaging youth between 15 and 25 years of age with MDD in a multimodal exercise intervention plus usual care to evaluate the magnitude of impact on psychological, physical fitness, and biomarker outcome. The exercise program involved a single session of motivational interviewing to enhance adherence, followed by a 1-h exercise session three times a week for 12 weeks. Depression assessment was done at 12 weeks by the Beck Depression Inventory, the mean scores of which showed a decrease from 31.9 ± 9.1 to 13.1 ± 10.1 (Cohen *d* effect size = 1.96). The positive impact highlighted by this study motivates recognizing the use of exercise as a powerful adjunct tool (Adriana et al., 2020). The prevalence of child and adolescent mental illness, including all mental disorders, was found to at 13.4% (Polanczyk et al., 2015). The updated World Health Organization (WHO) estimates on mental disorders identified the prevalence to be 22.1% (depression, bipolar disorder, schizophrenia, posttraumatic stress disorder (PTSD), and anxiety) at any time point among conflict-affected populations. The age-standardized and mean-comorbidity-adjusted prevalence was 13% for mild forms and 4% for moderate forms (Charlson et al., 2019). MDD is the third greatest disease factor for disease burden the world over, and with the existing psychosocial and pharmacological intervention, the rate of relapse of the quality and functioning of life among affected has been recorded to be impaired (Rapaport et al., 2005). Scientific reports have demonstrated moderate to higher intensity exercise as an effective adjunct treatment for improving depressive symptoms (Lawlor and Hopker, 2001; Daley, 2008; Schuch et al., 2016). The association between depression and quality of life has been well established, and even with pharmacological treatments, less than 50% on adequate dose experience significant clinical response (Sinyor et al., 2010). The underlying mechanism of action involving exercise as an intervention for anxiety and depression includes regulation in the production of the brain-derived neurotropic factor, hypothalamic pituitary adrenal axis, neuroinflammation, oxidative stress, D-β-hydroxybutyrate, and the GSK3β/β-catenin pathway. The HUNT cohort study involving a healthy cohort of 33,908 adults was followed up for 11 years and it identified regular leisure-time physical activity (LTPA) to reduce the incidence of depression (Harvey et al., 2018). Further, postadjustment for confounders, the population attributable fraction suggested that when the relationship is assumed to be causal, 12% of the future cases of depression becomes preventable by engaging in at least 1 h of PA every week, thus proving that LTPA of any intensity becomes protective against future depression (Manger and Motta, 2005). Aerobic exercise has also been studied in relation to symptoms of PTSD. Studies that examined the impact of a 12-week aerobic exercise program involving 30 min of jogging/walking between 60 and 80% maximum heart rate indicated a significant reduction in the symptom of PTSD. Another study involving 33 PTSD-affected were subjected to 2 weeks of stationary biking aerobic exercise of six sessions, and 89% of the participants reported significant reductions in severity after 2 weeks (Fetzner and Asmundson, 2014). With reference to studies on depression, a dose-dependent relation between exercise and depression scores have been found. One such study assessed the benefit of low-frequency exercise involving one aerobic session/week compared with high-frequency exercise involving three to five aerobic sessions/week and found a significant reduction in depression scores among participants in the latter group (Legrand and Heuze, 2007). Thus, promoting exercise in primary care centers, based on guideline recommendations, can provide scientifically proven benefits for a wide range of mood disorders and work as an effective adjunct for symptom management.

## Impact of Genetics on Exercise Benefits

**FIGURE 1:**
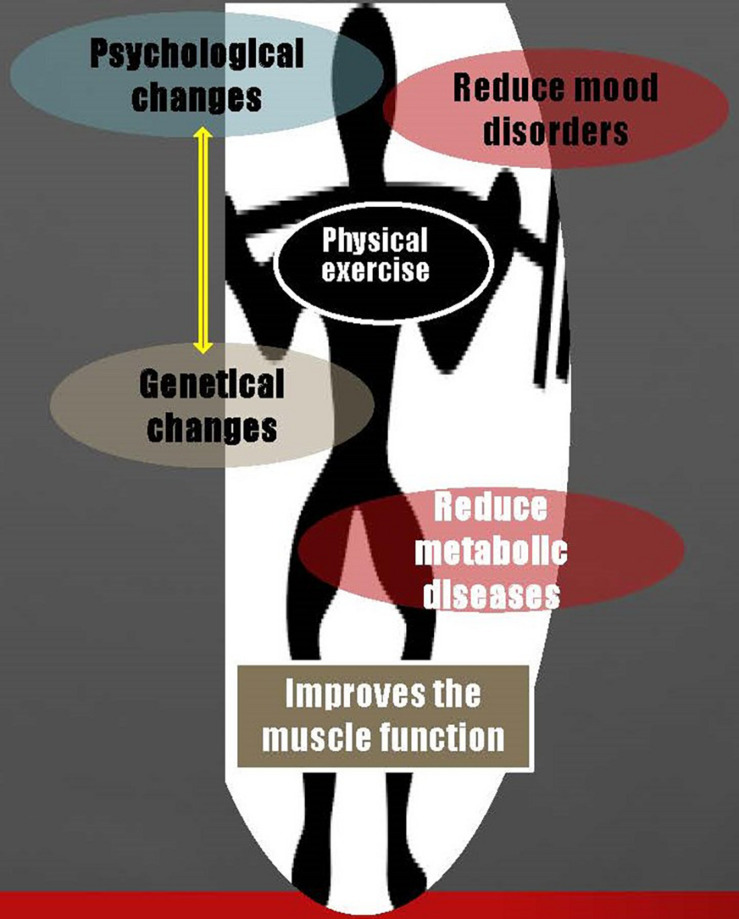
Physical exercise influences physiological, psychological, and genetical changes, which results in producing various benefits, including preventing metabolic and mood disorders.

The physiological as well as psychological impact of different forms of exercise and physical exercise has been associated with key genetic markers, which can modulate the outcome to a great extent (Figure 1). Scientifically established relation exists between risk genetic markers and their impact on attenuation by exercise regimen tailored as per impact linked genetics. To cite an example, one study among Taiwanese adults assessed the impact of aerobic exercise and badminton on levels of high-density cholesterol and its relation with the genetic variant rs328 in the lipoprotein lipase gene. This study involving 3,742 men and 4,071 women between the ages of 30 and 70 were subjected to no exercise, aerobic exercise, and badminton. Individuals engaged in the aerobic and the badminton exercise group exhibited higher levels of good cholesterol, high-density lipoproteins (HDL), and were also carriers of the rs328 genotypes (Nassef et al., 2020). Studies have also investigated the impact of genetic variants in leptin (*LEP*) and *LEP* receptor (*LEPR*) on habitual PA, and the body composition response to a unilateral upper body resistance training (RT) program. The variants rs2167270, rs1137100, rs1137101, rs1805096, and rs8179183 were studied. Carriers of the GG genotype of rs2167270 exhibited more kcal per week in vigorous-intensity PA and sports recreation compared with “A” allele carriers. In case of RT carriers of the *LEPR* “G” allele for variant rs1137101 exhibited greater gain in arm muscle and subcutaneous fat volume (Walsh et al., 2012). The fat mass and obesity-associated gene (*FTO*) is the first and widely studied obesity loci identified through genome-wide association studies. The variant rs9939609 has been widely reported, wherein each additional minor allele increases the risk of obesity by 20–30% (Frayling et al., 2007). Studies have correlated the impact of PA to attenuate obesity risk through *FTO* variant rs9939609. One such metaanalysis study, involving 45 studies on adults and nine on children, identified the minor allele “A” of rs9939609 to increase odds of obesity by 1.23-fold/allele. Further, the risk was also shown to be attenuated by PA, wherein the risk odds reduced to 1.22/allele, when compared with the inactive group with odds risk as 1.30/allele, thus exhibiting a risk reduction of 27% among the physically active (Kilpeläinen et al., 2011).

Aerobic exercise benefits on physiology among sedentary adults have also been evaluated with genetic markers. One such study involved participants in a 30-min submaximal aerobic exercise session and found two variants in the *FTO* gene, *viz* rs8044769 and rs3751812, to change positively during exercise (Karoly et al., 2012). The *CREB1* gene variants, *viz* rs2253206 and rs2360969, were linked to change in body temperature during exercise and with maximal oxygen capacity (VO_2_ max). The variant rs1379659 (*SLIT2* gene) and rs1935881 (*FAM5C* gene) were linked to changes in norepinephrine during exercise, whereas the *OPRM1* variant rs1799971 was linked to changes in norepinephrine, lactate, as well as the rate of perceived exertion during exercise. This highlights the impact of genetic markers in determining the outcome and benefit of aerobic exercise (Cagnin et al., 2019). Studies have also identified heritability to affect VO_2_ max response to exercise training by 47% (Jones et al., 2016). More than a dozen genetic variants have been linked to exercise-related traits and outcomes, and this paved way for the development of genetics-based algorithms for personalized training programs. One such study report highlighted an algorithm that facilitated the achievement of better results in response to high- and low-intensity RT program by predicting the potential of the athlete for power and endurance by studying 15 genetic markers linked to performance. This algorithm development included two studies involving athletes from different sports and soccer players who were subjected to 8-week low- and high-intensity RT with genetically matched and mismatched. The athletes in the matched group exhibited a significant increase in countermovement jump (CMJ; *p* = 0.0005) and aerobic 3-min cycle test (Aero3; *p* = 0.0004), whereas those in the mismatched group hardly exhibited any improvement. Further, among the soccer players, the matched group exhibited better improvement in CMJ and Aero3 (*p* < 0.0001). The frequency of nonresponders in this study was found to be 82% from the mismatched group. This reiterates the effectiveness of genetically-tailored exercise programs (Jones et al., 2016). Study literature till date reports on the presence of 36 genetic markers from mitochondrial DNA, Y chromosome, as well as autosomal genes to be linked to elite athlete status, whereas 39 genetic markers from 19 genes and mitochondrial DNA have been linked to interindividual variability in response to endurance/strength training (Ahmetov and Rogozkin, 2009). These findings highlight the significance and need to include genetic signature analysis when utilizing exercise as an intervention for risk, disease, as well as treatment management in known cases.

## Conclusion

The benefits of long-term exercise, which include better endurance capacity, stamina, as well as improved oxygen supply to the muscles, have been well documented. However, the kind of benefit that can be induced to alter the genetic status of the individual, including genetic markers are less reported. Genetic markers can also impact the body temperature during exercise, making the entire experience of training either comfortable or unpleasant. Thus, there is an overwhelming amount of scientific evidence which has gathered around the benefits of exercise, which has become a pressing need from the 21st century when the belief on the value of exercise started waning, and that spiked up the era of lifestyle and noncommunicable ailments.

## Author Contributions

YW and KA conceived and wrote this manuscript and approved the final version of the manuscript.

## Conflict of Interest

The authors declare that the research was conducted in the absence of any commercial or financial relationships that could be construed as a potential conflict of interest.

## Publisher’s Note

All claims expressed in this article are solely those of the authors and do not necessarily represent those of their affiliated organizations, or those of the publisher, the editors and the reviewers. Any product that may be evaluated in this article, or claim that may be made by its manufacturer, is not guaranteed or endorsed by the publisher.
